# Atrial Fibrillation Associated Chromosome 4q25 Variants Are Not Associated with PITX2c Expression in Human Adult Left Atrial Appendages

**DOI:** 10.1371/journal.pone.0086245

**Published:** 2014-01-22

**Authors:** Shamone R. Gore-Panter, Jeffery Hsu, Peter Hanna, A. Marc Gillinov, Gosta Pettersson, David W. Newton, Christine S. Moravec, David R. Van Wagoner, Mina K. Chung, John Barnard, Jonathan D. Smith

**Affiliations:** 1 Department of Cellular and Molecular Medicine, Cleveland Clinic, Cleveland, Ohio, United States of America; 2 Department of Molecular Medicine, Cleveland Clinic Lerner College of Medicine of Case Western Reserve University, Cleveland, Ohio, United States of America; 3 Cleveland Clinic Lerner College of Medicine of Case Western Reserve University, Cleveland, Ohio, United States of America; 4 Department of Thoracic and Cardiovascular Surgery, Cleveland Clinic, Cleveland, Ohio, United States of America; 5 Department of Molecular Cardiology, Cleveland Clinic, Cleveland, Ohio, United States of America; 6 Department of Cardiovascular Medicine, Cleveland Clinic, Cleveland, Ohio, United States of America; 7 Department of Quantitative Health Sciences, Cleveland Clinic, Cleveland, Ohio, United States of America; University of Miami School of Medicine, United States of America

## Abstract

Atrial Fibrillation (AF), the most common sustained arrhythmia, has a strong genetic component, but the mechanism by which common genetic variants lead to increased AF susceptibility is unknown. Genome-wide association studies (GWAS) have identified that the single nucleotide polymorphisms (SNPs) most strongly associated with AF are located on chromosome 4q25 in an intergenic region distal to the *PITX2* gene. Our objective was to determine whether the AF-associated SNPs on chromosome 4q25 were associated with *PITX2c* expression in adult human left atrial appendages. Analysis of a lone AF GWAS identified four independent AF risk SNPs at chromosome 4q25. Human adult left atrial appendage tissue was obtained from 239 subjects of European Ancestry and used for SNP analysis of genomic DNA and determination of *PITX2c* RNA expression levels by quantitative PCR. Subjects were divided into three groups based on their history of AF and pre-operative rhythm. AF rhythm subjects had higher *PITX2c* expression than those with history of AF but in sinus rhythm. *PITX2c* expression was not associated with the AF risk SNPs in human adult left atrial appendages in all subjects combined or in each of the three subgroups. However, we identified seven SNPs modestly associated with *PITX2c* expression located in the introns of the *ENPEP* gene, ∼54 kb proximal to *PITX2*. *PITX2c* expression in human adult left atrial appendages is not associated with the chromosome 4q25 AF risk SNPs; thus, the mechanism by which these SNPs are associated with AF remains enigmatic.

## Introduction

Atrial Fibrillation (AF), a complex cardiac arrhythmia, is the most common sustained arrhythmia encountered in clinical practice and affects over 2.3 million Americans and millions more worldwide [1]. AF is associated with a 2-fold increase in mortality and 4- to 5-fold increased risk for stroke, resulting in significant cost to the healthcare system [1], [2]. Previous genome wide association studies (GWAS) have found that the strongest single nucleotide polymorphisms (SNPs) associated with AF are located on chromosome 4q25, with the AF odds ratio for the minor allele ranging from ∼1.4 to 2.0 [3]–[5]. Among the AF-associated SNPs on chromosome 4q25, several independent risk variants have been identified [6]. These SNPs are located in an intergenic region of chromosome 4q25 with the closest gene, *PITX2*, located ∼171 kb proximal to the most significant SNP identified in the earliest GWAS, rs2200733 [5].

*PITX2*, a member of the bicoid class of homeobox genes, is expressed in many organs throughout the body, including the heart and brain [7]. There are 3 major human isoforms, *PITX2a*, *PITX2b*, and *PITX2c*. *PITX2c* is the only isoform expressed in the left atrium [8], and it has been shown to play a critical role in left/right asymmetry during development in the heart [9]. Using RNAseq, we have previously shown that *PITX2c* is expressed in the human left atrial appendage and not in the right atrial appendage [8]. Although homozygous *Pitx2c* deficiency in mice results in embryonic lethality [10], [11], hemizygous *Pitx2c* +/− mice are viable and susceptible to pacing-induced atrial arrhythmia, making it an attractive candidate as an AF-causal gene [12].

Many SNPs identified in GWAS for complex traits such as anthropomorphic measures and common diseases are located in intergenic regions and thus it is challenging to determine the mechanism for the observed associations. The overriding hypothesis is that these SNPs affect complex traits by regulating the expression of nearby genes, thus these SNPs may be classified as cis-acting expression quantitative trait loci (eQTLs). It is also challenging to determine which are the causal SNPs, as the index SNPs may not be causal but in linkage disequilibrium (LD) with causal SNPs. Recent findings from the ENCODE project show that most of the GWAS SNPs, or SNPs in strong LD with the GWAS SNPs, are within regulatory regions. These regions were identified due to their location within a DNAseI hypersensitive region or within a site in which histone modification or transcription factor binding indicates regulatory activity, as determined by chromatin-immunoprecipitation and next generation sequencing [13]. We hypothesized that the four SNPs independently associated with AF in the chromosome 4q25 region would be associated with *PITX2c* expression. To study this, we used SNP arrays to obtain genotypes and quantitative RT-PCR (qRT-PCR) to measure *PITX2c* expression in left atrial appendages obtained from 239 subjects of European ancestry, including 40 samples from subjects with no history of AF. We found that these AF-associated SNPs were not associated with *PITX2c* mRNA expression in adult left atrial appendages in all subjects combined, or in the subgroup of subjects with no history of AF. However, we identified several SNPs in introns of the *ENPEP* gene, on the opposite side of the *PITX2* gene relative to the location of the AF-associated SNPs, which were modestly associated with *PITX2c* mRNA expression levels. Thus, the mechanism of the AF-associated SNPS on 4q25 remains unknown.

## Materials and Methods

### Ethics Statement

All patients provided informed consent for use of discarded atrial tissue. Prior to 2008 verbal consent was obtained and documented in the medical records in a process approved by the Cleveland Clinic Institutional Review Board (IRB). From 2008 onward and for donor tissues, patients provided separate IRB-approved written informed consent. The IRB approved the studies included in this report. Subjects were not consented for genetic information sharing, thus the SNP data cannot be loaded to dbGAP.

### Genome Wide Association Studies (GWAS) and Identification of Independent AF Associated SNPs at Chromosome 4q25

Descriptions of the cohorts used in the Cleveland Clinic lone AF (LAF) GWAS, genotyping, and quality control and filtering of samples and SNPs were previously described. [3]. Here we include an additional 111 Caucasian LAF cases from the Cleveland Clinic Lone Atrial Genebank, all typed on the Illumina Hap610 chip, giving a total of 607 LAF cases and 2956 population controls for GWAS and fine mapping analysis. Maximum likelihood logistic regression was used to estimate the association between odds of LAF and each of the 479,618 filtered genotyped SNPs. SNPs were coded as allele dosages. The logistic model included sex and four principal components of genetic sharing, all of which were associated with LAF. LAF-SNP association p-values were adjusted for any residual population stratification using the genomic control method [14]. Odds ratios of LAF were estimated for each SNP.

Results from our LAF GWAS were used to find independent LAF-SNP associations in the 4q25 region. Starting with SNP rs2200733, which was previously reported as the strongest AF-associated SNP in a GWAS [5] and had the largest odds ratio in our LAF GWAS, we searched for additional SNPs in the vicinity of rs2200733 (150 SNPs over 1 Mb) that were independently associated with LAF using a forward step-wise approach. SNPs were added until the conditional p-value, adjusting for all SNPs chosen in the step-wise search, was less than 0.01 with Bonferroni correction for 150 SNPs (p<6.67×10^−5^).

### Human Left Atrial Tissue Processing

Human left atrial appendage tissues obtained from elective surgery were snap frozen in liquid nitrogen and stored at −80°C until RNA extraction. AF history, type of AF, structural heart disease, demographics, and other clinical data were collected in a research database and a prospectively collected database of all cardiac surgeries (The Cardiovascular Information Registry) maintained by the Department of Cardiothoracic Surgery. Subjects were categorized as “lone AF” if they had a history of AF and did not have coronary artery disease or valvular disease. AF rhythm status was determined by review of electrocardiograms obtained prior to surgery. 16 left atrial tissue specimens were obtained from non-failing donor hearts not used for transplant. These hearts were perfused with cardioplegia prior to explant and processed in the same manner as hearts used for organ transplant. As with the surgical specimens, donor tissue samples were snap frozen in liquid nitrogen and kept at −80°C until RNA extraction.

### Genomic DNA Isolation and SNP Microarray

25–50 mg of left atrial appendage tissue was used to extract DNA. The tissue, in one mL of DNAzol® (Invitrogen,), was homogenized (PowerGen700, Fisher Scientific) with sterile Omni Tip Disposable Generator Probes (Omni International,). DNA was isolated from the homogenate following the manufacturer’s protocol. The DNA pellet was resuspended in 20 µl of 10 mM Tris buffer (pH 7.4) and the DNA concentration was measure with a NanoDrop ND-1000 Spectrophotometer (Thermo Fisher Scientific Inc.), diluted to 100 ng/µl and stored at −20^o^C until use. The DNA was genotyped using Illumina Hap550v3 and Hap610-quad SNP microarrays. Only directly genotyped SNPs were used in this study.

### RNA Isolation

50–100 mg of left atrial appendage tissue was used to extract RNA. The tissue, in one ml of TRIzol® (Invitrogen), was homogenized with a sterile Omni Tip Disposable Generator Probes. RNA was isolated from the homogenate following the manufacturer’s protocol. The RNA pellet was dried and resuspended in 80 µl of DEPC water and the concentration was measured with the NanoDrop ND-1000 Spectrophotometer and stored at −80^o^C.

### cDNA Preparation

1 µg of purified RNA was added to 4 µl of Superscript® Vilo™ mastermix (Invitrogen) and water added to bring the reaction volume to 20 µl. The reaction was run in an ABI themocycler at 25°C for 10 min, 42°C for 120 min and 85°C for 5 min with a 4°C hold temp. After completion, 5 µl of the newly synthesized cDNA was diluted with 90 µl of nuclease free water and stored at −20^o^C until further use.

### Quantitative Reverse Transcriptase-polymerase Chain Reaction (qRT-PCR)

An Eppendorf Epmotion 5070 robotic pipettor was used to prepare the working and reaction plates. To prepare the master mix for each sample, 12.5 µl of the TaqMan® gene expression master mix (Applied Biosystems) was used along with 1.25 µl of the custom designed *PITX2c* primer/probe set (Table 1, obtained from IDT) or *SHOX2* primer/probe set (assay number Hs00243203_m1 from Applied Biosystems) and the primer limited cardiac actin (*ACTC1)* primer/probe mix (assay number Hs00606316_m1 from Applied Biosystems). PITX2c expression was also normalized to primer limited cyclophilin A (*PPIA*) primer/probe mix (assay number Hs04194521_s1 from Applied Biosystems). This 15 µl mix was pipetted into individual wells of a 96-well working plate. Using the robot, 10 µl of the diluted cDNA was added. 5 µl of the total mixture from the working plate was pipetted in triplicate to a 384-well assay plate. PCR was performed in a Bio-RAD CRX qRT-PCR machine that had been calibrated for our FAM and VIC fluorescent probes. Thermal cycling was performed with a hot-start at 95°C for 10 minutes, followed by 40 cycles of 95°C for 15 seconds and 60°C for 60 seconds. Delta C(t) values for *PITX2c* and *SHOX2* expression levels were calculated relative to *ACTC1* expression, and the ΔΔCT method was used to compare expression among samples [15], yielding log2 based expression values.

**Table 1:** PITX2c TaqMan primer and probe set.

Identifier		Sequence
PITX2c Forward Primer		5′-GCG GTT CCT CTG GAA AGT GG-3′
PITX2c Reverse Primer		5′-GCA CAC CAT CTC CGA CAC CT-3′
Probe*		5′/56-FAM/CCC GGA GGC/ZEN/CGC AGA GAA AGA TAA/3IABkFQ/−3′

* FAM fluorophore with internal ZEN and 3′ IOWA BLACK FQ quencher modification.

### *PITX2c* Expression Analysis

Relative log_2_ gene expression levels were corrected for plate and batch effects using three standardized atrial RNA samples on each plate. Relative expression levels were fit to an additive linear model including age, gender, donor/surgical sample, atrial fibrillation history and pre-operative rhythm status, using the R statistical program. Differences in *PITX2c* expression among the rhythm groups was determined by non-parametric ANOVA.

### *PITX2* eQTL Analysis

For the four AF susceptibility SNPs on chromosome 4q25, relative *PITX2c* expression levels were fit to an additive linear model including age, gender, donor/surgical sample, AF history, pre-operative rhythm status, and genotype using the R statistical program. Analysis was performed on all 239 samples. For regional eQTL analysis in the chromosome 4q25 locus, 169 assayed SNPs from the Illumina SNP microarray +/−500 Kb from the *PITX2* gene were tested for association with *PITX2c* expression levels using R. This analysis was confined to 223 samples, excluding the 16 donors, where all clinical information was known. Relative *PITX2c* expression levels were fit to an additive linear model including age, gender, history of coronary artery disease (CAD), history of mitral valve disease (MVD), history of hypertension, body mass index (BMI), atrial fibrillation history, pre-operative rhythm status, and genotype using the R statistical program. Significance for the regional eQTL p-values was determined by deviation from the expected values using a quantile-quantile (QQ) plot. Additional analysis and plotting were performed with GraphPad Prism software. Power analyses for eQTL studies were performed in R using a linear model test at f2 values of 0.02, 0.15, and 0.35.

## Results

### Patient Characteristics of 239 Adult Left Atria Tissue

**Figure 1:**
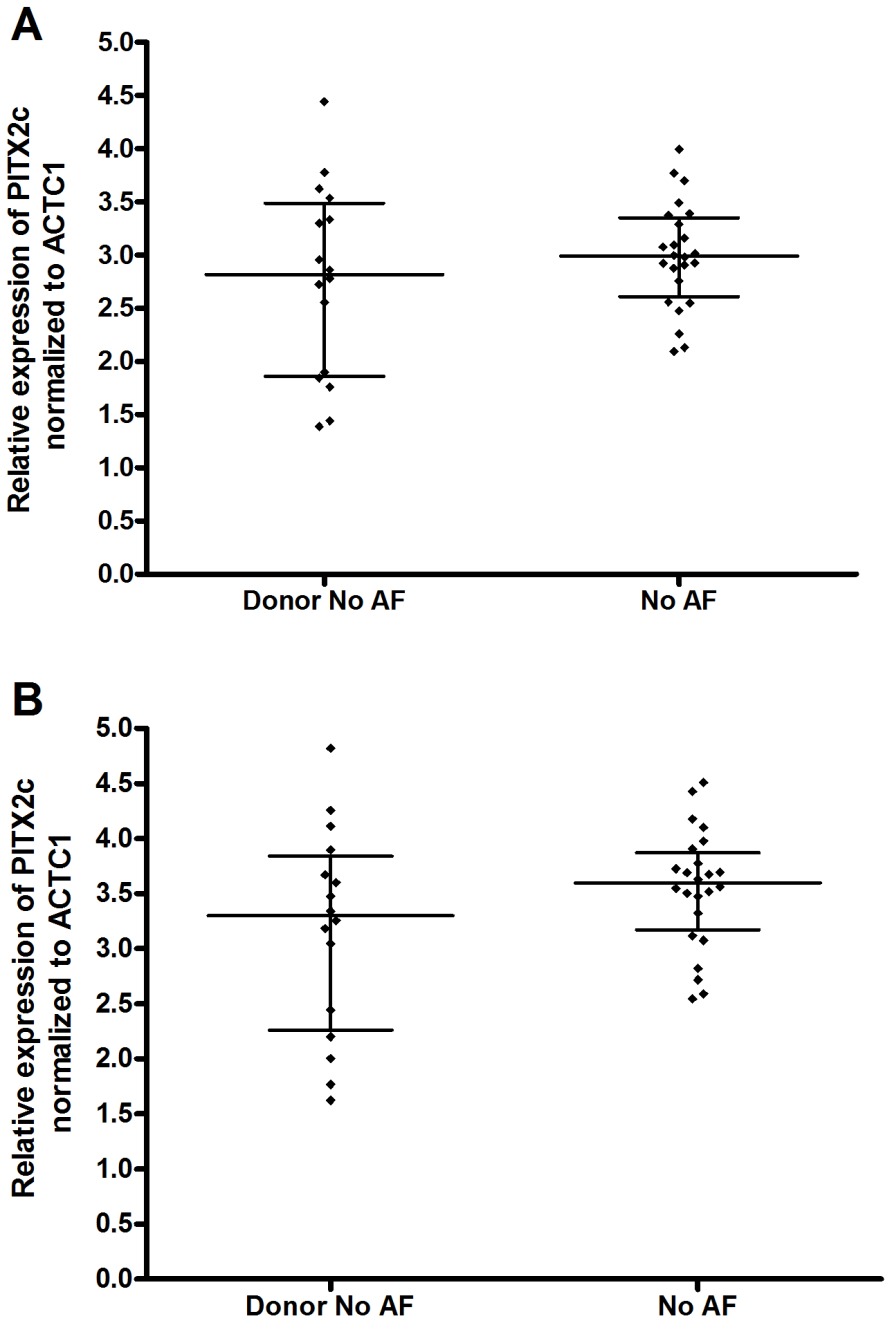
Adjusted and unadjusted expression of *PITX2c* in human left atrial appendages in AF controls. Log2 *PITX2c* expression, normalized to *ACTC1*, in the 16 donor and 24 surgical No AF samples uncorrected (A), or after correction for age and sex (B). There was no significant difference in *PITX2c* expression between these groups by non-parametric Mann-Whitney t-test. Individual values are shown along with median and interquartile range.

223 left atrial appendages were obtained during cardiac surgery from subjects of European ancestry. 16 additional were obtained from transplant donors that were not used for transplantation. Samples were divided into three groups based on their history of AF and their preoperative rhythm status: no history of AF (No AF, N = 40); history of AF in sinus rhythm at time of sample collection (AF/SR, N = 78); and history of AF in AF rhythm at time of sample collection (AF/AF, N = 121). 24 of the 40 No AF subjects were in surgery to treat other cardiac conditions, while the remaining 16 donor samples were assumed to have no history of AF. There was no significant difference in PITX2c expression between the 24 No AF surgical samples and the 16 No AF donor samples before or after correction for sex and age (Figure 1). However, there was a trend for lower *PITX2c* expression in the donor samples. Thus, in subsequent analyses we combined these 40 subjects into one No AF group, and corrected expression for donor status. We examined if the AF/rhythm status groups were associated with sex, age, BMI, and history of hypertension, CAD, and MVD; although, for the No AF group we had to exclude the donor samples for association with BMI, hypertension, CAD, and MVD, since this data was not available for these samples (Table 2). Females constituted 23% of the cohort with no statistically significant differences in sex among the rhythm groups (p = 0.11). Age (range 16–86 years old) was associated with rhythm status with the AF/AF group being the oldest and the AF/SR group being the youngest (p-value  = 0.040). The four subjects <31 years old were all in the No AF group. BMI for our cohort ranged from 17.8–46.9, with a trend for the highest BMI in the AF/AF group and the lowest in the No AF group (p-value  = 0.064). History of hypertension was present in 51% of the subjects, with a trend towards a higher frequency in the No AF group (p = 0.085). History of CAD was present in 34% of the subjects and trended higher in those with No AF (50%, p = 0.17). History of MVD was present in 49% of the subjects and trended higher in the No AF group (70%, p = 0.076). Among the 199 subjects with a history of AF, 35 had lone AF. These subjects were equally represented in the AF/SR and AF/AF groups. However, the lone AF subjects were significantly younger with a median age of 56 (45–61 interquartile range) than the other AF subjects with a median age of 63 (interquartile range 44–70, p<0.0001 by Mann Whitney two tailed t-test).

**Table 2:** Left atrial appendage surgical and donor patient characteristics.

Patient Characteristics	Total N = 239	No AF n = 40 (16 donors),17%	AF/SR n = 78, 33%	AF/AF n = 121, 50%	P-Value
**Sex, Female, %**	23%	35%	24%	19%	0.11#
**Age (years)** *	61 (53, 69)	62 (51,71)	59 (52, 65)	64 (55, 70)	0.040$
**BMI (kg/m^2^)** * &	27.8 (24.7, 31.6)	26.5 (23.4,28.3)	27.0 (24.0, 31.4)	28.2 (25.2, 32.2)	0.064$
**Hypertension, %** &	51%	60%	41%	56%	0.085#
**CAD, %** &	34%	50%	29%	34%	0.17#
**MVD, %** &	49%	70%	53%	43%	0.076#
**Lone AF, %**	15%	0%	17%	18%	0.78##

* Median (interquartile range).

& Not including donors, for which this information not available.

# p-value by chi-square test.

$ p-value by Kruskal Wallis nonparametric ANOVA.

## p-value by chi-square comparing only AF/SR and AF/AF groups.

### Four Independent SNPs at Chromosome 4q25 Associated with AF

**Figure 2:**
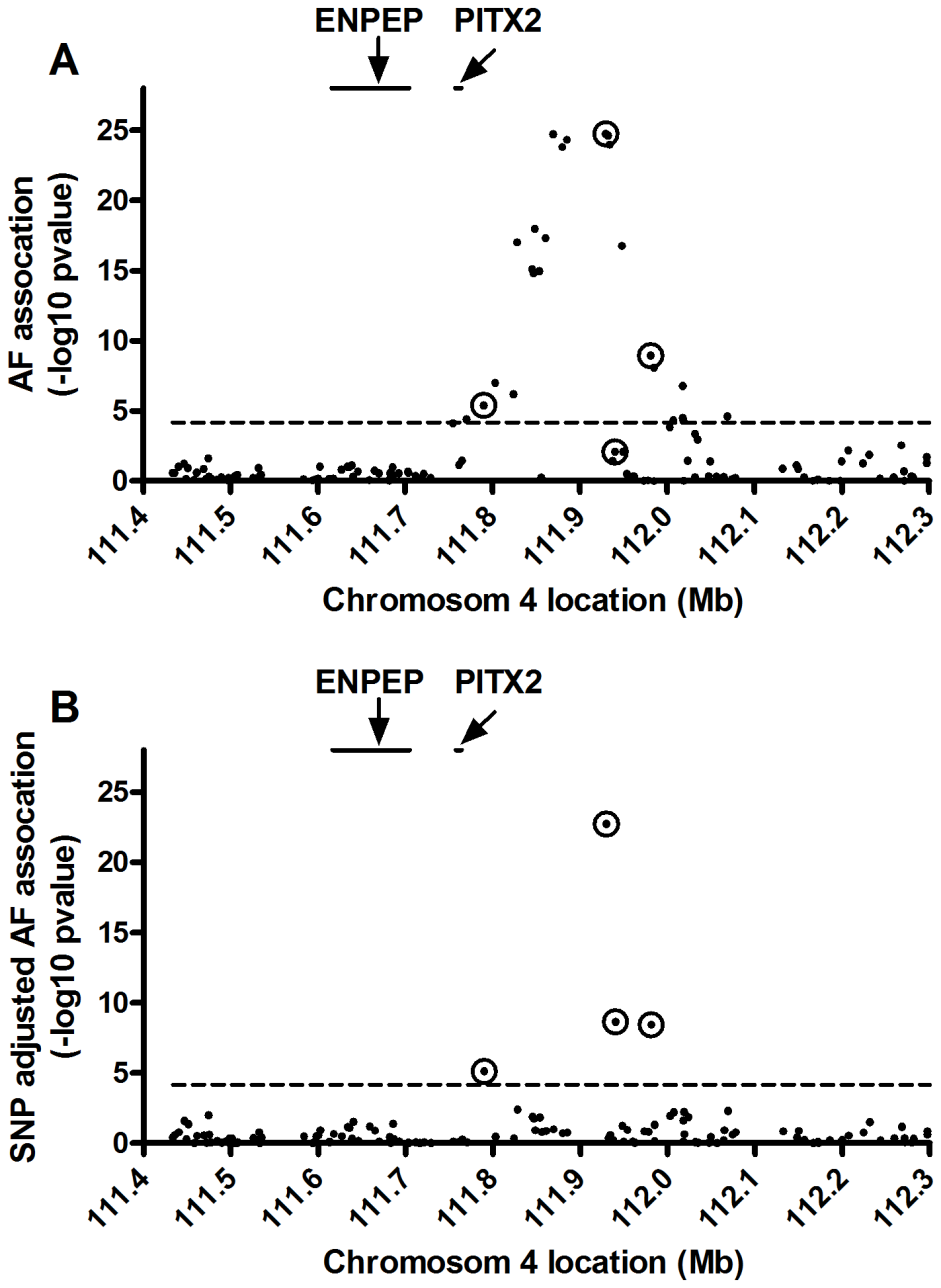
Identification of four SNPs independently associated with AF at the 4q25 locus in the Cleveland Clinic Lone AF GWAS. **A.** AF associations of all genotyped SNPs in the 4q25 locus using the marginal model described in Table 3. The locations of the PITX2c and ENPEP genes are shown above. The dashed line is the Bonferroni corrected level of significance for the 150 SNPs tested at p = 0.01. **B.** Since many of the SNPs in this region are in LD with each other, the AF associations were recalculated after adjustment for the remaining significant SNPs using the full model described in Table 3. The four independently associated SNPs are in the center of the circles in both panels.

Prior GWAS and meta-analyses have shown that the 4q25 locus has the strongest association with AF. We performed a GWAS using 607 Cleveland Clinic lone AF cases and 2956 Illumina population controls, which confirmed that the strongest locus associated with AF resides at chromosome 4q25. Figure 2A shows the fine map of this AF association at 4q25. We performed a conditional analysis to identify independent SNPs associated with AF in this region. Four SNPs were found to be independently associated with AF at the locus-wide significance threshold of p<6.67×10^−5^ (Figure 2B and Table 3). Two of these SNPs, rs2200733 and rs3853445, had previously been found to be independently associated with AF [6]. The minor alleles of three of these SNPs are associated with increased risk, while the minor allele of rs385445 was associated with decreased risk for AF. The most highly AF-associated SNP, rs2200733, had an odds ratio of 2.47. These four SNPs are in weak linkage disequilibrium with each other (Table 4), showing that four separate haplotype blocks in this locus are associated with AF.

**Table 3:** 4q25 SNPs Independently Associated with AF.

			Marginal Model Results		Full Model Results	
SNP	Position on Chr 4	MAF	OR	P-Value	OR	P-Value
rs2200733	111929618	0.17	2.47	1.80E-25	2.46	1.80E-23
rs10033464	111940210	0.10	1.32	8.34E-03	1.99	2.26E-09
rs3853445	111980936	0.25	0.59	1.14E-09	0.59	3.65E-09
rs1448818	111789672	0.25	1.42	3.87E-06	1.44	7.66E-06

Results from logistic regression fits of the following 2 models.

Marginal Model for each SNP: History of lone AF using as covariates Sex +4 principal components of genetic sharing.

Full Model for each SNP: History of lone AF using as covariates Sex +4 principal components of genetic sharing+other 3 SNPs shown on table.

**Table 4:** Linkage disequilibrium (r^2^) of 4 risk/independently associated AF SNPs in 4q25 Region.

	rs2200733	rs3853445	rs1448818	rs10033464
**rs2200733**	1			
**rs3853445**	0.0018	1		
**rs1448818**	0.047	0.016	1	
**rs10033464**	0.0064	0.110	0.018	1

### Covariates Affecting *PITX2c* Expression Levels

**Figure 3:**
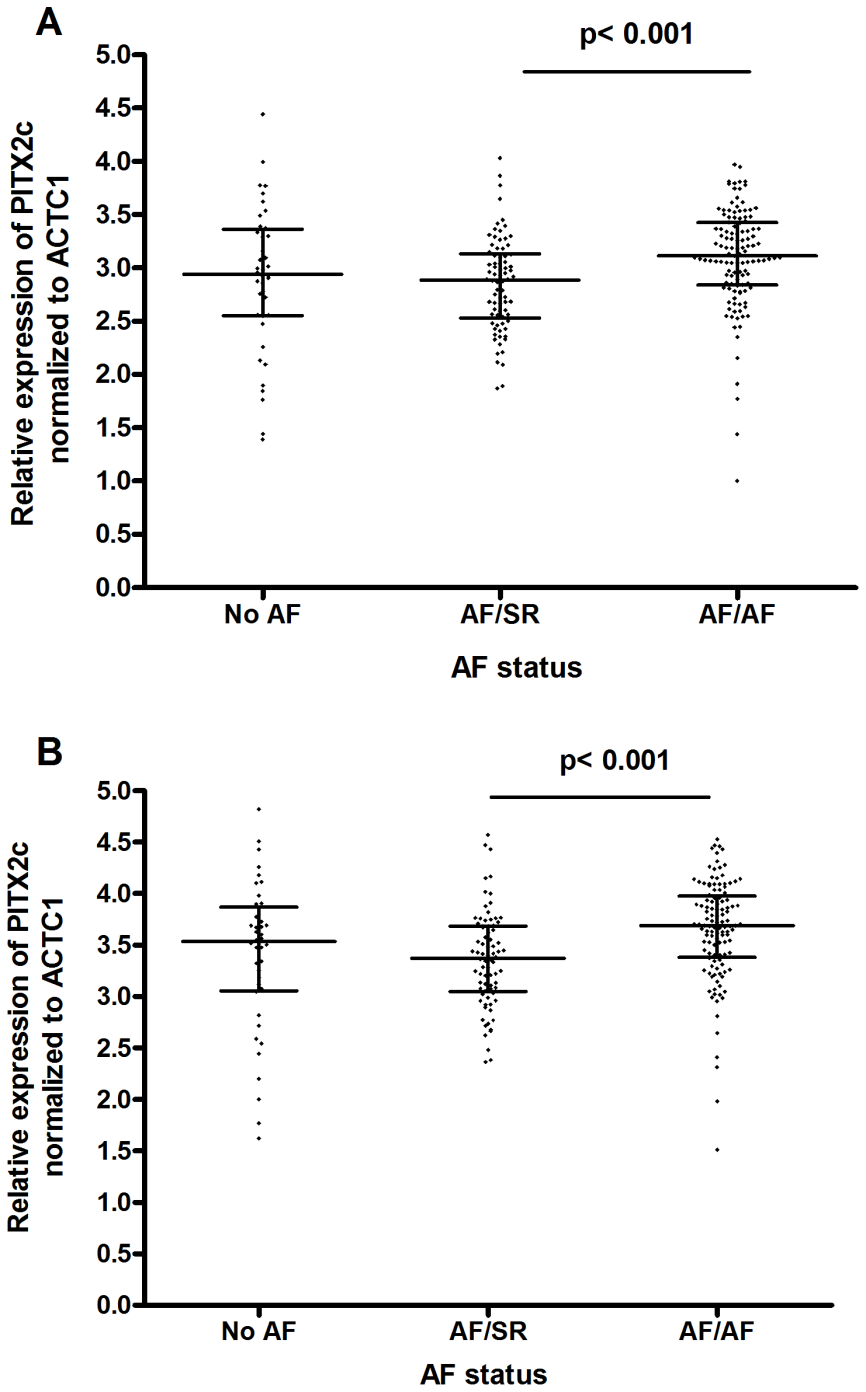
*PITX2* expression was associated with AF history and rhythm status. **A.** Unadjusted levels of *PITX2c* in the different AF history/rhythm groups revealed a U-shaped relationship with increased AF-status. *PITX2c* median levels were 17% higher (antilog2 transformed) in the AF/AF vs. AF/SR groups (p<0.001). **B.** *PITX2c* levels adjusted for sex and age. The U-shaped relationship was maintained with *PITX2c* median levels 24% higher (antilog2 transformed) in the AF/AF vs. AF/SR groups (p<0.001). Individual subject data is shown along with the median and interquartile range.

Expression of *PITX2c* normalized to ACTC1 was measured by qRT-PCR in RNA derived from the left atrial appendages. Log_2_ *PITX2c* expression levels were examined in the different AF history/rhythm groups (Figure 3). Surprisingly, there was a U-shaped relationship with increased AF disease status, such that *PITX2c* expression was higher in subjects with no history of AF, lower in AF/SR subjects, and higher again in AF/AF subjects, with this difference highly significant (p = 2×10^−4^ by non-parametric Kruskal-Wallis ANOVA) (Figure 3A). Dunn’s ANOVA post-test indicated that the only significant difference among the three groups was between the AF/SR and AF/AF groups (p<0.001), although the effect size was moderate with 17% higher median *PITX2c* expression levels (antilog_2_ transformed) in the AF/AF vs. AF/SR groups. After adjusting for sex and age, the relationship between *PITX2c* expression and AF history/rhythm was maintained (p<1×10^−4^ overall), with 24% higher median *PITX2c* expression (antilog2 transformed) in the AF/AF vs. AF/SR groups (p<0.001, Figure 3B).

We then looked at each covariate individually for an association with *PITX2c* expression. Age, history of hypertension, and history of MVD were all significantly associated with *PITX2c* expression at p<0.05; and, history of CAD had a trend with p = 0.07. The strongest covariate associated with *PITX2c* expression was rhythm status in those with a history of AF, such that *PITX2c* expression in the AF/SR group was significantly different from those in the AF/AF group (p = 9.14×10^−4^, Table 5). However in a multivariate model adjusting for all covariates, the only covariates significantly associated with *PITX2c* expression were age (p = 5.79×10^−3^, Table 5) and rhythm status in those with a history of AF, such that *PITX2c* expression in the AF/SR group was significantly different from those in the AF/AF group (p = 4.59×10^−3^, Table 5). In the multivariate analysis the log_2_ effect size and direction on *PITX2c* expression for each covariate is indicated by the β coefficient.

**Table 5:** Multivariate Model for PITX2c expression.

Covariate	Unadjusted p-value	Adjusted p-value#	b for adjusted p-value
Sex	0.20	0.22	0.10
Age	1.13E-03	5.79E-03	0.01
BMI&	0.18	0.79	1.57E-03
Hypertension&	0.01	0.24	0.08
CAD&	0.07	0.82	0.02
MVD&	0.03	0.16	−0.10
No history of AF/donors	0.25*	0.19$	−0.15
Donors only	0.185*	0.94$	−0.013
AF/SR	9.14E-04*	4.59E-03	−0.21

* vs. AF/AF group.

# adjusted for all covariates vs. AF/AF group, unless marked otherwise.

& Not including donors, for which this information not available.

$ adjusted only for age and sex vs. AF/AF group.

### *PITX2c* cis-eQTLs

**Figure 4:**
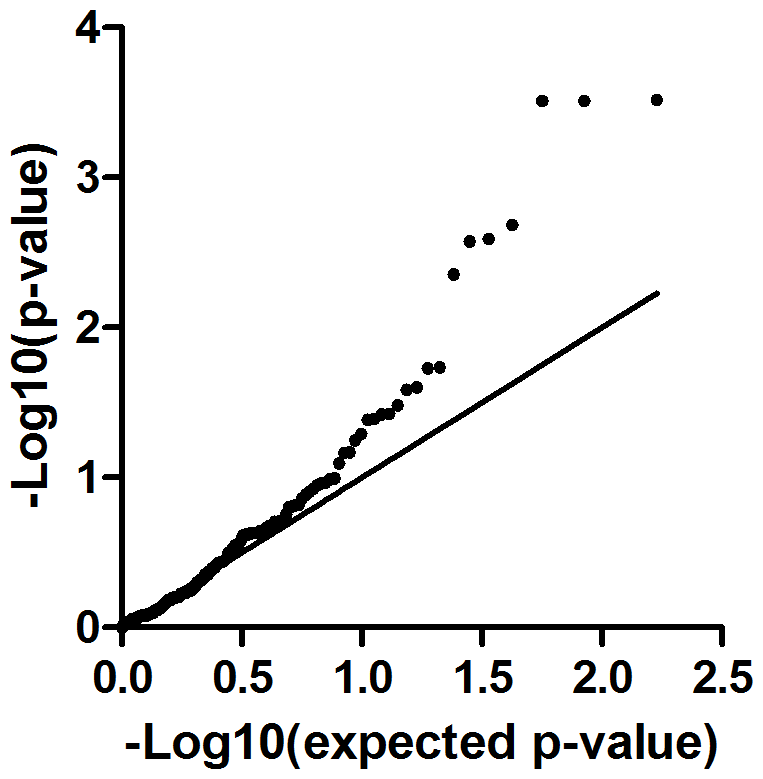
QQ plot of *PITX2c* eQTLs in the 4q25 region. Seven SNPs were far above the expected p-value range for association with adjusted PITX2c levels.

**Figure 5:**
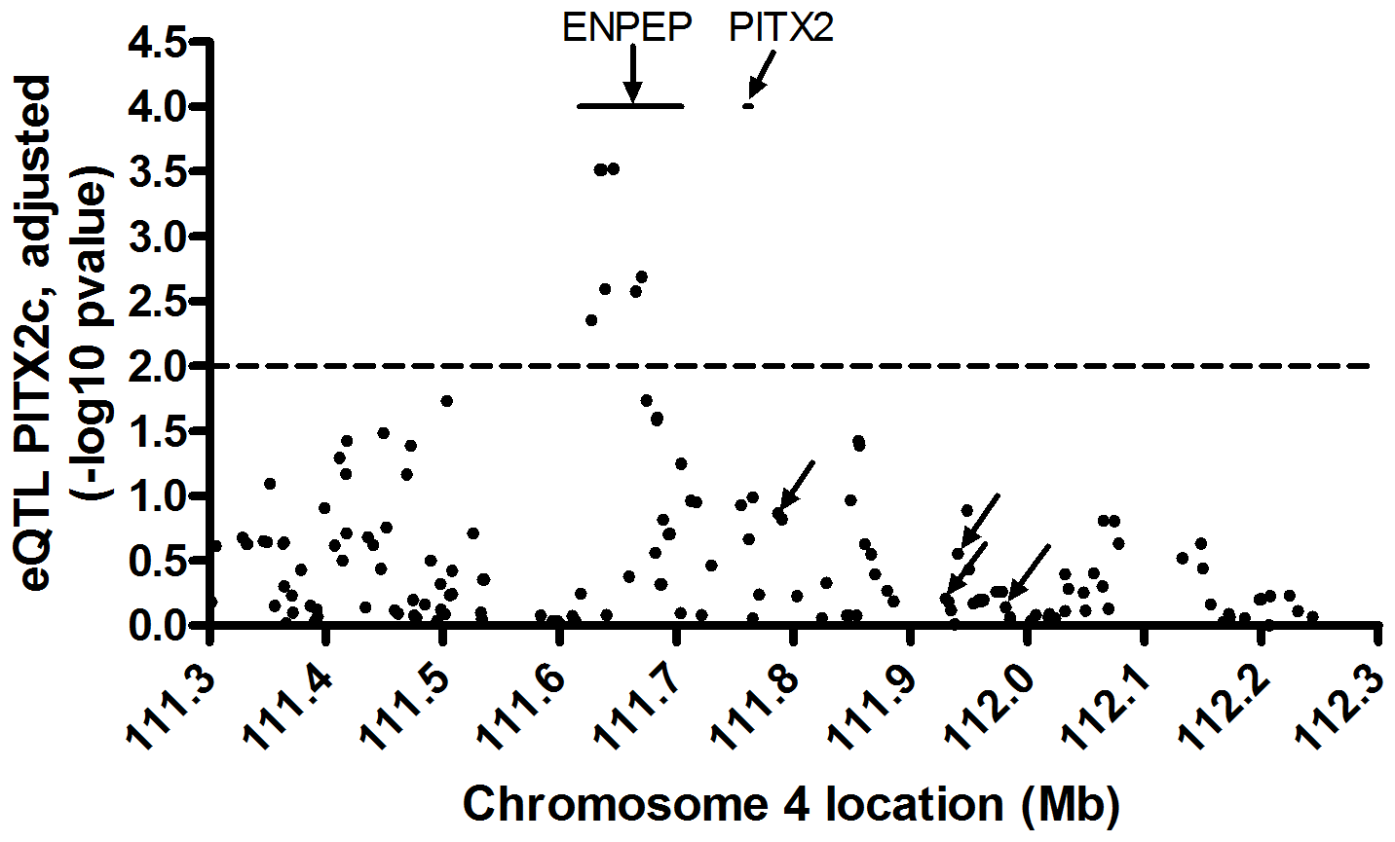
*PITX2c* eQTLs for SNPs in the 4q25 region. All genotyped SNPs +/−500 kb from *PITX2c* were evaluated for *PITX2c* eQTLs. The four AF associated SNPs are shown with arrows. Seven SNPs within introns of the *ENPEP* gene were associated with expression of *PITX2c* (p<0.01).

We assessed the four independent AF SNPs on chromosome 4q25 for association with the expression of *PITX2c*, thus probing whether these SNPs serve as cis-eQTLs for *PITX2c*. None of these SNPs were associated with *PITX2c* levels when normalized to ACTC1, a cardiomyocyte-specific reference gene (p≥0.15, Table 6) or when normalized to PPIA, a reference gene expressed in all cell types (p≥25, Table 7). After adjusting *PITX2c* expression for sex, age, donor status, and AF history/rhythm there still was no association of these four SNPs with expression of *PITX2c* (Tables 6, 7). We were well powered to detect SNPs effects on the expression of *PITX2c* in the 239 human left atrial appendages, with 99% power to observe a 15% log_2_ fold change in *PITX2c* expression (Table 8). In order to determine if we could uncover any cryptic associations in any rhythm subgroup, we evaluated each of the three AF history/rhythm groups to determine if there were any significant associations of these four SNPs with unadjusted or adjusted *PITX2c* expression. No significant associations were discovered at p<0.05 in any of the subgroups (Table 9). Among the 40 No AF subjects, we had moderate power to detect SNP effects on *PITX2c* expression, with 38% and 78%power to observe a 15% and 35% log_2_ fold change in PITX2c expression, respectively (Table 8). Upon analysis of genotyped SNPs over the region +/−500 kb from *PITX2c*, there were seven SNPs outside of the expected p-value range in QQ plots that were associated with the adjusted *PITX2c* expression levels (Figure 4). These SNPs were associated with *PITX2c* expression at p<0.01, with the top SNP at p = 3.0×10^−4^ (Figure 5, Table 9). These SNPs are located within introns of the *ENPEP* gene, which is proximal on chromosome 4 to the *PITX2* gene; while the AF associated SNPs are distal to *PITX2*. These seven SNPs are all in LD with each other and thus represent one haplotype block (r^2^ from 0.292 to 1, Table 10).

**Table 6:** Chr. 4 AF risk SNPs not associated with PITX2c expression normalized to ACTC1.

SNP	location	PITx2c p-value uncorrected	R of PITX2c uncorrected and 95%CI	PITX2c p-value phenotype corrected*
rs2200733	111929618	0.90	0.0084 [−0.12–0.14]	0.72
rs3853445	111980936	0.68	0.027 [−0.10*0.15]	0.96
rs1448818	111789672	0.40	0.0548 [−0.073–0.18]	0.37
rs10033464	111940210	0.15	−.0936 [−0.23–0.034]	0.09

* corrected for sex, age, donor status, and AF history/rhythm.

CI, confidence interval.

**Table 7:** Chr. 4 AF risk SNPs not associated with PITX2c normalized to PPIA expression.

SNP	location	PITx2c p-value uncorrected	PITX2c p-value phenotype corrected*
rs2200733	111929618	0.25	0.39
rs3853445	111980936	0.63	0.97
rs1448818	111789672	0.40	0.33
rs10033464	111940210	0.81	0.51

* corrected for sex, age, donor status, and AF history/rhythm.

**Table 8:** Power Analysis for PITX2c expression.

PITX2c log2 effect	Power in No AF cohort (N = 40)*	Power in full cohort (N = 239)#
0.02	8%	30%
0.15	38%	99%
0.35	78%	99.99%

* Covariates were age, sex, donor status, and SNP yielding 3 degrees of freedom.

# Covariates were age, sex, donor status, 3 AF history/rhythm at surgery groups, and SNP yielding 6 degrees of freedom.

**Table 9:** P-values for AF SNPs association with PITX2c expression in individual AF history/rhythm groups.

	No AF (N = 40)		AF/SR (N = 78)		AF/AF (N = 121)	
SNP	Uncorrected	Corrected*	Uncorrected	Corrected#	Uncorrected	Corrected#
rs2200733	0.267	0.513	0.189	0.110	0.978	0.886
rs3853445	0.522	0.451	0.863	0.998	0.374	0.448
rs1448818	0.832	0.917	0.157	0.119	0.789	0.652
rs10033464	0.422	0.122	0.237	0.302	0.518	0.592

* corrected for age, sex, and donor status.

# corrected for age, sex, BMI, and history of hypertension, CAD, and MVD.

**Table 10:** Linkage disequilibrium (r^2^) between significant PITX2c eQTL SNPs located in the ENPEP Gene.

SNP name	rs11731078	rs2348427	rs2881913	rs639194	rs16997154	rs1448808		rs6533524	PITX2p-value		Chr 4 bp	Intron		
rs11731078	1									3.04E-04	111645790		3rd	
rs2348427	0.571	1								3.07E-04	111633848		3rd	
rs2881913	0.571	1	1							3.07E-04	111635731		3rd	
rs639194	0.915	0.505	0.505	1						2.06E-03	111669772		10th	
rs16997154	0.298	0.522	0.522	0.245	1					2.56E-03	111638646		3rd	
rs1448808	0.44	0.802	0.802	0.485	0.501		1			2.67E-03	111665036		10th	
rs6533524	0.341	0.68	0.68	0.292	0.55		0.519	1		4.42E-03	111626900		1st	

### *PITX2c* Expression is Inversely Correlated with *SHOX2* Expression

**Figure 6:**
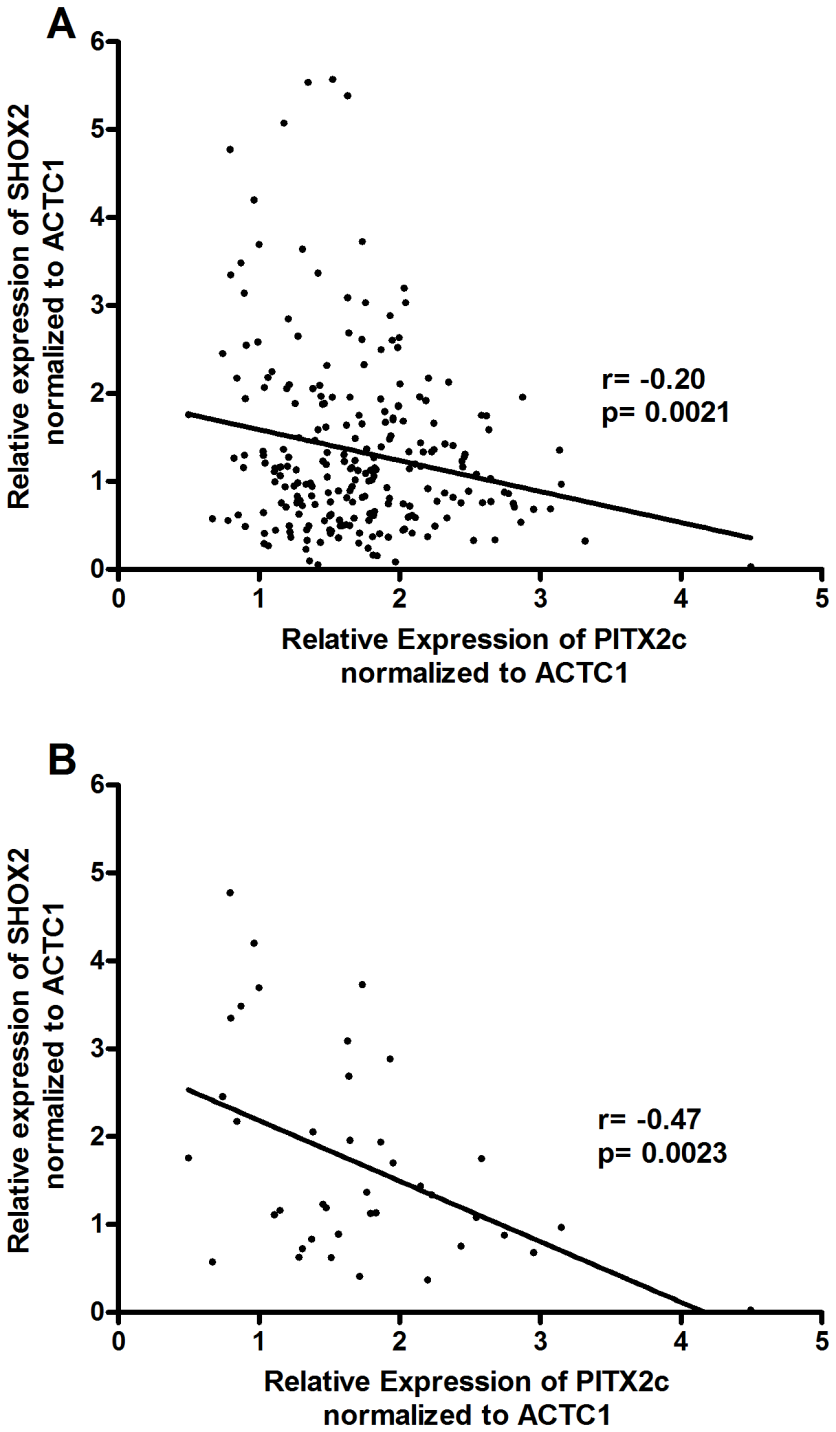
Correlation analysis of *PITX2c* with *SHOX2* expression in human left atrial appendages. **A.** *SHOX2* expression was inversely correlated with *PITX2c* expression in all 239 subjects (r = −0.20 p = 0.0021) **B.** Among the three AF status/rhythm groups, the inverse correlation between *SHOX2* and *PITX2c* was only found in the No AF subgroup (r = −0.47, p = 0.0023).

*SHOX2* is a transcription factor that plays a role during embryonic development promoting the formation of the sinoatrial node [16]. In mice, Shox2 is repressed by Pitx2, as demonstrated by higher *Shox2* mRNA levels in *Pitx2*−/+ vs. *Pitx2*+/+ mouse hearts [12]. Bioinformatic analysis identified a conserved Pitx2c recognition element in the 2nd intron of the *Shox2* gene [12]. Reporter gene transfections confirmed that Pitx2c directly represses gene expression via this recognition element in the *Shox2* gene [12]. We measured *SHOX2* expression by qRT-PCR to examine whether there was a correlation between *PITX2c* and *SHOX2* expression in the human left atrial appendages. We found an inverse correlation between *PITX2c* and *SHOX2* expression in these samples (r = −0.20, p = 0.0021, Figure 6a). Additionally we further analyzed the results based on AF history/rhythm status and found an even stronger inverse correlation within the subgroup with no history of AF (r = −0.47, p = 0.0023, Figure 6b), while this correlation was not significant in the AF/SR and AF/AF groups.

## Discussion

Analysis of our Cleveland Clinic Lone AF GWAS data revealed four independent AF associated SNPs in the chromosome 4q25 region, the strongest being the previously identified rs2200733 SNP, which had an odds ratio of 2.47 (p = 1.8×10^−25^). This odds ratio is higher than those previously reported of 1.4 to 2 [3]–[5], which we attribute to the use of a lone AF cohort. Genetic susceptibility in the lone AF cohort may play a larger role in AF pathogenesis than in a mixed cohort of AF subjects, in which many of the cases may be secondary to structural heart diseases such as CAD and MVD. We then utilized 239 human adult left atrial appendages to determine whether these four independent AF SNPs were associated with expression of *PITX2c*, the gene closest to these SNPs, although rs2200733 is 150.6 kb distal to the *PITX2* gene on chromosome 4. While these SNPs were not associated with expression of *PITX2c* in adult human left atrial appendage tissues, we did find seven SNPs in introns of *ENPEP*, proximal to the *PITX2* gene, that were associated with *PITX2c* expression.

*PITX2c* expression was associated with AF rhythm status, such that there was significantly increased expression of *PITX2c* in the AF/AF groups compared to the AF/SR group. This was surprising because we predicted that *PITX2c* expression might decrease with AF burden, since *Pitx2c*−/+ mice are susceptible to pacing-induced atrial arrhythmia [12]; yet, the highest *PITX2c* expression we observed was in the AF/AF group. A prior study using surgically obtained atrial tissues from only 5 No AF and 5 matched AF subjects found that *PITX2c* expression was lower in 4/5 of the AF subjects than the No AF controls [17]; however, the current study has a much larger sample size, allowing a multivariate regression model to compare *PITX2c* expression levels among the various rhythm groups. It is known that AF, especially permanent AF, is associated with structural, contractile, and electrophysiological remodeling [18], [19]. It has been suggested that AF induces a cellular adaptation through de-differentiation into a more fetal-like cell phenotype that promotes cell survival during stress [19]–[21]. Using a mouse model where lacZ was knocked into the *Pitx2* gene, Wang et al. demonstrated that *Pitx2* expression is highest in the left atrium in 3-day old mice, moderate in the left atrium of 42-day old mice, and only expressed in a few residual cells of the left atrium in 1-year old mice [12]. Thus, we speculate that the fetal-like reprogramming associated with increased AF burden might lead to the reactivation of *PITX2c* expression in previously non-expressing cells and explain why we observed the highest *PITX2c* levels in the AF/AF group. Overall, we found that *PITX2c* expression in the subjects with no history of AF was not statistically different than its expression in the subjects with a history of AF; thus, *PITX2c* expression levels in adult left atrial appendages cannot be used to distinguish AF cases from controls.

We predicted that the AF-associated SNPs at chromosome 4q25 might regulate left atrial expression of *PITX2c*; however, our results were not consistent with this hypothesis in the adult left atrial appendages. This negative finding may have resulted from examining *PITX2c* expression at the wrong time and/or wrong place. For example, it might be possible to identify the effect of these SNPs on *PITX2c* expression in left atrial tissue from neonates, where *PITX2c* expression may be expressed highly in all left atrial cardiomyocytes. However, we speculate that the effects of these SNPs on expression may be lost in the residual cells that retain *PITX2c* expression in adult left atria, where epigenetic modifications may mask the SNP effects. Furthermore, *Pitx2* expression in the lacZ knock-in mouse was very high in the pulmonary vein region of 3-day old mice [12]; and the pulmonary vein region is the target of therapeutic ablation that often suppresses AF. Thus, it is possible that the AF-associated SNPs regulate *PITX2c* expression in the pulmonary vein, but not in the left atrial appendage. Another possibility is that the AF-associated SNPs actually control the expression of other adjacent protein coding or non-coding genes that are involved in AF pathogenesis, even though *PITX2* is the closest gene.

One approach that might be used to identify functional activity of the regions containing the four AF-associated SNPs would be the identification of enhancer/silencer activity using reporter gene transfection studies, or using transgenic mice or zebrafish. However, a caveat to this strategy is that heart enhancers have been shown to be weakly conserved even between humans and mice, thus limiting the efficacy of animal models that could be used to investigate heart enhancer functional activity [22].

We identified several SNPs, located in introns one, three, and ten of the *ENPEP* gene that were associated with expression of *PITX2c* in human adult left atrial appendages. The *ENPEP* gene codes for glutamyl aminopeptidase or aminopeptidase A (APA). APA is a homodimeric type II membrane-bound protease [23] that converts angiotensin (Ang) II to Ang III by cleaving the N-terminal aspartic acid residue of Ang II [24]. Ang III is a potent agonist of the angiotensin receptor type 1, and there is evidence that it may have a more important role than Ang II in the brain in sustaining hypertension in the spontaneous hypertensive rat [25], [26]. However, the *Enpep* knockout mouse displays hypertension, arguing that APA also plays a systemic role in the catabolism of angiotensin activity [24]. A human GWAS has shown that a common SNP in *ENPEP*, rs6825911, is associated with hypertension in East Asians [27]. This SNP is in weak LD with rs16997154 in Asians (r^2^ = 0.321 in the Asian populations, HapMap release 2.2), which we found to be an eQTL for *PITX2c* expression in subjects of European ancestry. However, the SNPs in *ENPEP* that we identified as *PITX2c* eQTLs in adult left atria were not associated with lone AF in our GWAS (Figure 2) or for AF in prior GWAS. Thus, we found no connection between *PITX2c* expression, its association with SNPs in the *ENPEP* gene [3], and susceptibility to AF.

In our cohort, we confirmed that *PITX2c* expression was inversely correlated with *SHOX2* expression as seen in a mouse study [12]; however when analyzing the data based on rhythm status, only the group with no history of AF remained significant. We found this surprising, because we would have expected to observe a significant inverse correlation in all subgroups. This suggest that tissue remodeling and/or epigenetic changes in disease conditions may override the direct effect of *PITX2c* on *SHOX2* expression, thus rendering the no AF history group better for analysis of transcription factor associations with their downstream targets.

Our study was limited by the restricted availability of atrial appendages from subjects with no history of AF. The No AF group consisted of 40 subjects, 24 subjects undergoing cardiac surgery for medical reasons plus 16 donor samples that were not used for transplantation. Within this No AF group, we still found no association of any of the four AF associated SNPs with *PITX2c* expression; however, we had only moderate power to find eQTLS in this subgroup (Table 8). In general, fewer surgical samples are available currently, as catheter ablation has become more common than surgical ablation for AF treatment, and because left atrial appendage resections have become less common with the introduction of left atrial appendage exclusion devices. In conclusion, we found no evidence that the AF risk SNPs at chromosome 4q25 are associated with *PITX2c* expression in adult left atrial appendages, and we suggest that these risk SNPs may be regulating *PITX2c* and/or other nearby genes during development or in the pulmonary vein region.
